# Pulmonary Carcinoid Surface Receptor Modulation Using Histone Deacetylase Inhibitors

**DOI:** 10.3390/cancers11060767

**Published:** 2019-06-03

**Authors:** Rachael E. Guenter, Tolulope Aweda, Danilea M. Carmona Matos, Jason Whitt, Alexander W. Chang, Eric Y. Cheng, X. Margaret Liu, Herbert Chen, Suzanne E. Lapi, Renata Jaskula-Sztul

**Affiliations:** 1 Department of Surgery, School of Medicine, University of Alabama at Birmingham, Birmingham, AL 35233, USA; rguenter@uab.edu (R.E.G.); danileacm@sanjuanbautista.edu (D.M.C.M.); jwhitt@uabmc.edu (J.W.); awchang@uab.edu (A.W.C.); hchen@uabmc.edu (H.C.); 2 Department of Radiology, University of Alabama at Birmingham, Birmingham, AL 35233, USA; taweda1@uabmc.edu (T.A.); lapi@uab.edu (S.E.L.); 3 San Juan Bautista School of Medicine, Caguas, PR 00726, USA; 4 College of Pharmacy, University of North Texas Health Science Center, Fort Worth, TX 76107, USA; eric.cheng@unthsc.edu; 5 Department of Biomedical Engineering, University of Alabama at Birmingham, Birmingham, AL 35233, USA; mliu@uab.edu

**Keywords:** somatostatin receptor, histone deacetylase inhibitor, neuroendocrine cancer, pulmonary carcinoid

## Abstract

Pulmonary carcinoids are a type of neuroendocrine tumor (NET) accounting for 1–2% of lung cancer cases. Currently, Positron Emission Tomography (PET)/CT based on the radiolabeled sugar analogue [^18^F]-FDG is used to diagnose and stage pulmonary carcinoids, but is suboptimal due to low metabolic activity in these tumors. A new technique for pulmonary carcinoid imaging, using PET/CT with radiolabeled somatostatin analogs that specifically target somatostatin receptor subtype 2 (SSTR2), is becoming more standard, as many tumors overexpress SSTR2. However, pulmonary carcinoid patients with diminished SSTR2 expression are not eligible for this imaging or any type of SSTR2-specific treatment. We have found that histone deacetylase (HDAC) inhibitors can upregulate the expression of SSTR2 in pulmonary carcinoid cell lines. In this study, we used a non-cytotoxic dose of HDAC inhibitors to induce pulmonary carcinoid SSTR2 expression in which we confirmed in vitro and in vivo. A non-cytotoxic dose of the HDAC inhibitors: thailandepsin A (TDP-A), romidepsin (FK228), suberoylanilide hydroxamic acid (SAHA), AB3, and valproic acid (VPA) were administered to promote SSTR2 expression in pulmonary carcinoid cell lines and xenografts. This SSTR2 upregulation technique using HDAC inhibitors could enhance radiolabeled somatostatin analog-based imaging and the development of potential targeted treatments for pulmonary carcinoid patients with marginal or diminished SSTR2 expression.

## 1. Introduction

The leading cause of cancer-related deaths worldwide is currently attributed to lung cancer, with pulmonary carcinoids accounting for 1–2% of all cases [1]. Pulmonary carcinoids are a type of neuroendocrine tumor (NET) with a five-year survival rate ranging from 40–90% [2]. Classification includes low-grade typical carcinoids (TC), intermediate grade atypical carcinoids (AC), and a rare pre-invasive carcinoid lesion known as diffuse idiopathic NE cell hyperplasia (DIPNECH) [1]. Positron Emission Tomography (PET)/CT using the radiolabeled sugar analogue [^18^F]FDG is used to diagnose and stage high grade, poorly-differentiated carcinoids such as ACs, but is suboptimal due to low metabolic activity in these tumors [3].

More recently, NET imaging using PET/CT with radiolabeled somatostatin analogs that specifically target somatostatin receptor subtype 2 (SSTR2) is becoming more standard as many NETs overexpress SSTR2 [4,5,6,7]. Indigenously, somatostatin is an anti-cancer neuropeptide, which is associated with the prevention of hormone and growth factor secretions that contribute to tumor growth, inhibition of tumor cell proliferation, and the induction of apoptosis mediated by the five different SST receptor subtypes (SSTR1-5) [8,9]. Clinically used somatostatin analogs have high affinities towards SSTR2, in addition to weaker affinities to SSTR3 and SSTR5 [10]. Most prominently, [^68^Ga]DOTATATE binds to SSTR2 with an order of magnitude higher in affinity than other [^68^Ga]-DOTA-peptides [10,11,12]. This suggests that a higher incidence and density of SSTR2 would be beneficial for imaging and treating patients [8]. However, only 34% TC, 71% of metastatic TC and 51% of AC showed a strong membrane presence of SSTR2. As the majority of pulmonary carcinoid patients have low or undetectable SSTR2 expression, the targeting might be inconsistent and peptide receptor radiotherapy (PRRT) can be limited only to patients with high level of SSTR2 [13]. Ultimately, well-differentiated, typical carcinoids (TC) can be imaged using PET/CT with radiotracers that target SSTR2, but poorly-differentiated, atypical carcinoids (AC) are only eligible for PET/CT using [^18^F]FDG, a technique shown to be insufficient for detection [14,15,16].

We have found that histone deacetylase (HDAC) inhibitors can increase the expression of SSTR2 in pulmonary carcinoid cell lines. Specifically, we have tested the FDA approved drugs: romidepsin (FK228), suberoylanilide hydroxamic acid (SAHA), valproic acid (VPA), in addition to a non-FDA approved analog of SAHA named AB3 and thailandepsin A (TDP-A), a naturally derived HDAC inhibitor isolated from the bacteria *Burkholderia thailandensis* E264 found in Thai rice fields [17,18]. Previous studies show that TDP-A has an anti-proliferative effect in various cancer cell lines at nanomolar concentrations and more specifically this compound may be a therapeutic agent against NETs by activating the Notch pathway [19,20,21,22].

This study was designed to test our hypothesis that the HDAC inhibitors can increase the presence of SSTR2 on the surface of pulmonary carcinoid cells to potentially improve SSTR2-based imaging and therapies. We performed in vitro studies and small animal PET/CT with [^68^Ga]DOTATATE to characterize alterations in SSTR2 expression after treatment HDAC-inhibiting compounds.

## 2. Results

### 2.1. Transcriptional and Translational Induction of SSTR2

**Figure 1:**
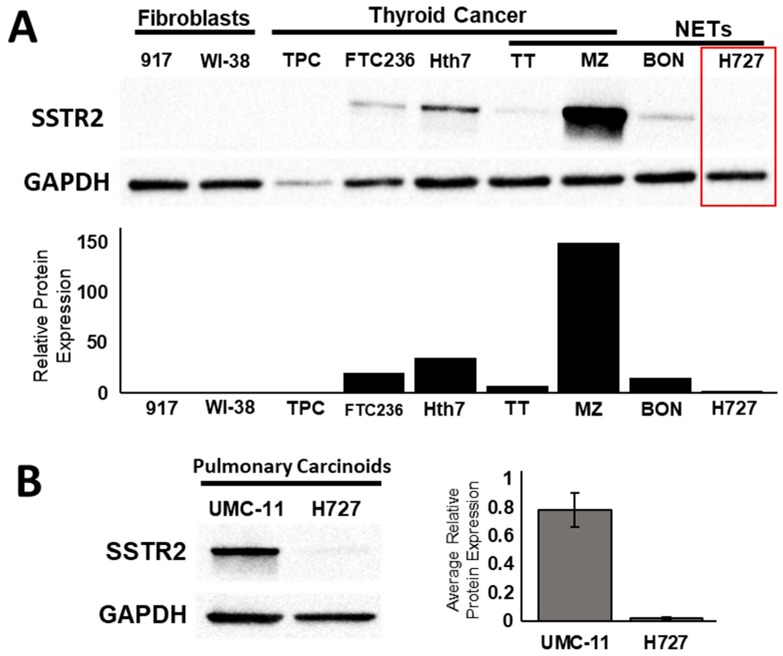
Basal levels of SSTR2 protein expression. (**A**) Basal expression level of SSTR2 in human fibroblast cell lines (917 and WI38), aggressive thyroid cancer cell lines (TPC, FTC236, and Hth7) and in neuroendocrine cancer cell lines (medullary thyroid cancer: TT and MZ; pancreatic NE cancer: BON; pulmonary carcinoid: H727). H727 cell line has the lowest basal expression of SSTR2 among all NET cells (Supplementary Figure S1). (**B**) A comparison of basal SSTR2 expression at the protein level between two pulmonary carcinoid cell lines: UMC-11 and H727 (Supplementary Figure S2). Error bars show standard deviation.

In vitro studies showed the upregulation of SSTR2 at the protein and mRNA level following treatment with HDAC inhibiting compounds. We measured the basal protein expression level of SSTR2 in human fibroblast cells (917 and WI38 cells), aggressive thyroid cancer cell lines (TPC, FTC236, and Hth7), and in various NET cell lines (medullary thyroid cancer: TT and MZ cells, pancreatic NE cancer: BON-1, and pulmonary carcinoid: H727) (Figure 1). The pulmonary carcinoid cell line H727 had the lowest basal expression of SSTR2 among all NET cells (Figure 1A, Figure S1). Relative to the loading control, the ratio of SSTR2 protein expression in H727 was 0.26, compared to 0.79 in TT, 2.21 in MZ-CRC-1, 0.54 in BON-1, 1.09 in Hth7, 0.82 in FTC236, 0.25 in WI-38 and 0.09 in 917. To compare the basal SSTR2 expression between to widely available pulmonary carcinoid cell lines, we performed a western blot and determined that the average protein expression in UMC-11 was about 68-fold higher than in H727 (Figure 1B, Figure S2). This result suggests that the UMC-11 cell line could represent a patient with a high level of SSTR2 expression and H727 could represent a patient with a minimal level of SSTR2 expression.

**Figure 2:**
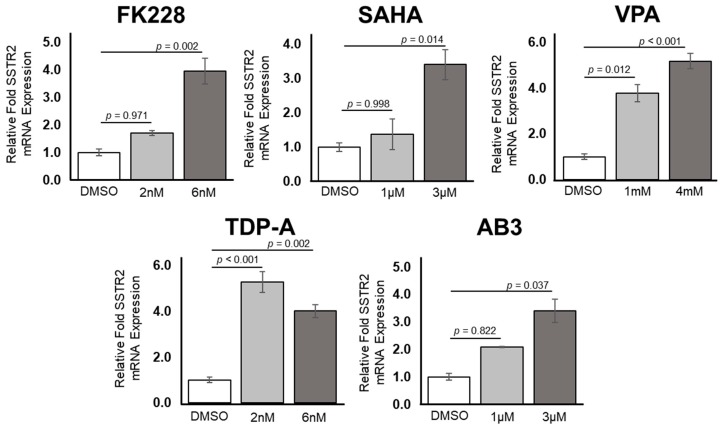
Upregulation of SSTR2 at the transcriptional level in H727, a pulmonary carcinoid cell line. Using two concentrations of five different histone deacetylase (HDAC) inhibitors, there is an increase in SSTR2 mRNA expression. There was a statistically significant increase after treatment with 6 nM romidepsin (FK228), 3 µM suberoylanilide hydroxamic acid (SAHA), 1 mM and 4 mM valproic acid (VPA), 2 nM and 6 nM thailandepsin A (TDP-A) and 3 µM AB3.

Upon treatment with increasing doses of five separate HDAC inhibitors, there were consistent increases in the expression of SSTR2 messenger RNA (mRNA) in the pulmonary carcinoid cell line H727 (Figure 2). An independent one-way ANOVA revealed a significant effect of treating H727 cells with HDAC inhibiting compounds F(10) = 14.240, *p* < 0.001. After administration of FK228, there was a significant increase in relative fold SSTR2 mRNA expression after a 6 nM dose (mean ± SEM, 4.01 ± 0.28, *p* = 0.002), but not a significant increase after a 2 nM dose (mean ± SEM, 1.72 ± 0.45, *p* = 0.971) when compared to a control treatment using DMSO. A similar trend was apparent after treatment with SAHA. There were increases in relative fold SSTR2 mRNA expression, where the increase was determined to be significant after a 3 µM dose of SAHA (mean ± SEM, 3.45 ± 0.46, *p* = 0.014), but not significant after a 1 µM dose of SAHA (mean ± SEM, 1.51 ± 0.44, *p* = 0.998). H727 cells treated with either 1 mM VPA (mean ± SEM, 3.80 ± 0.37, *p* = 0.012) or 4 mM VPA (mean ± SEM, 5.79 ± 0.34, *p* < 0.001) both had a significantly higher relative fold expression of SSTR2 mRNA compared to the DMSO treatment. Moreover, treatment with 2 nM TDP-A (mean ± SEM, 5.31 ± 0.45, *p* < 0.001), 6 nM TDP-A (mean ± SEM, 4.03 ± 0.57, *p* = 0.002), or 3 µM of AB3 (mean ± SEM, 3.43 ± 0.42, *p* = 0.037) showed a significantly higher relative fold inductions of SSTR2 at the mRNA level. A dose of 1 µM AB3 (mean ± SEM, 2.10 ± 0.02, *p* = 0.822) also showed a trend toward increased SSTR2 expression, but this increase was not found to be significant. All relative fold expression values were compared to a DMSO control treatment (mean ± SEM, 1.01 ± 0.12) and normalized to GADPH expression. In summary, transcription of the SSTR2 gene can be significantly increased with a 6 nM dose of FK228, a 3 µM dose of SAHA, both a 1mM and 4mM dose of VPA, both a 2 nM and a 6 nM dose of TDP-A and a 3 µM dose of AB3.

**Figure 3:**
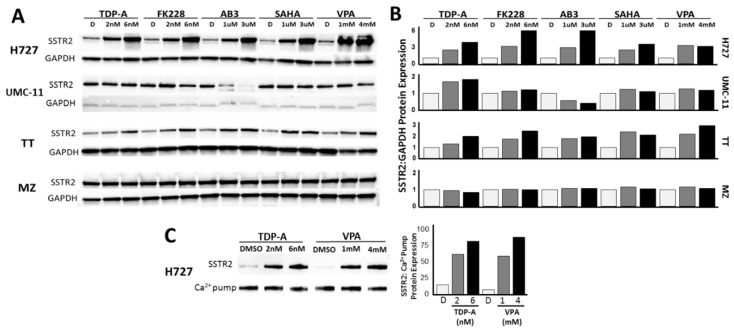
Increase in SSTR2 protein expression. (**A**) Upregulation of SSTR2 expression in NET cell lines by HDAC inhibitor treatment. The pulmonary carcinoid cell line, H727, showed the highest fold induction of SSTR2 (Supplementary Figure S3). (**B**) Ratio of SSTR2 protein expression relative to GADPH protein expression in NET cell lines after incubation with HDAC inhibitors (**C**) Upregulation of SSTR2 expression in the cell surface proteins of pulmonary carcinoid cell line H727 after TDP-A and VPA administration (Supplementary Figure S4).

To further investigate the ability of HDAC inhibitors to induce SSTR2 expression in pulmonary carcinoids in vitro, we assessed changes in the amount of SSTR2 protein after treatment (Figure 3, Figure S3). In agreement with the transcription level results, western blotting revealed increases in SSTR2 protein expression after treatment with increasing doses of five different HDAC inhibitors in the H727 cell line (Figure 3A, Figure S3A). Administration of 6 nM FK228 induced the highest relative fold expression of SSTR2 when compared to GAPDH expression. Each concentration of HDAC inhibitor-induced SSTR2 expression by at least 2.5-fold in the H727 cell line. However, there was a minimal increase of 1.8-fold in SSTR2 protein expression in the other pulmonary carcinoid cell line UMC-11, which had high basal expression (Figure 3A, Figure S3B). As a comparison to other NET cell lines, the effect of HDAC inhibitor treatment on SSTR2 protein incidence was also determined in medullary thyroid cancer cell lines: TT and MZ (Figure 3A, Figure S3C,D). Interestingly, a similar trend to the pulmonary carcinoid cell lines was observed. The TT cell line, with medium SSTR2 basal protein expression, showed increases in SSTR2 expression after treatment up to 3-fold, but the MZ cell line, with high basal SSTR2 expression, had only a maximum of 1.2-fold increase in the amount of detectable SSTR2 protein expression (Figure 3A, Figure S3D). To test to cell membrane presence of the observed SSTR2 overexpression, H727 cell surface proteins were isolated and probed for SSTR2 via western blot (Figure 3C, Figure S4). As shown in Figure 3C, treatment with 2 nM TDP-A, 6 nM TDP-A, 1 mM VPA, and 4 mM VPA all showed detectable increases in cell surface SSTR2 expression by at least 50%.

### 2.2. Detection of Functional SSTR2 Expression in Pulmonary Carcinoids

**Figure 4:**
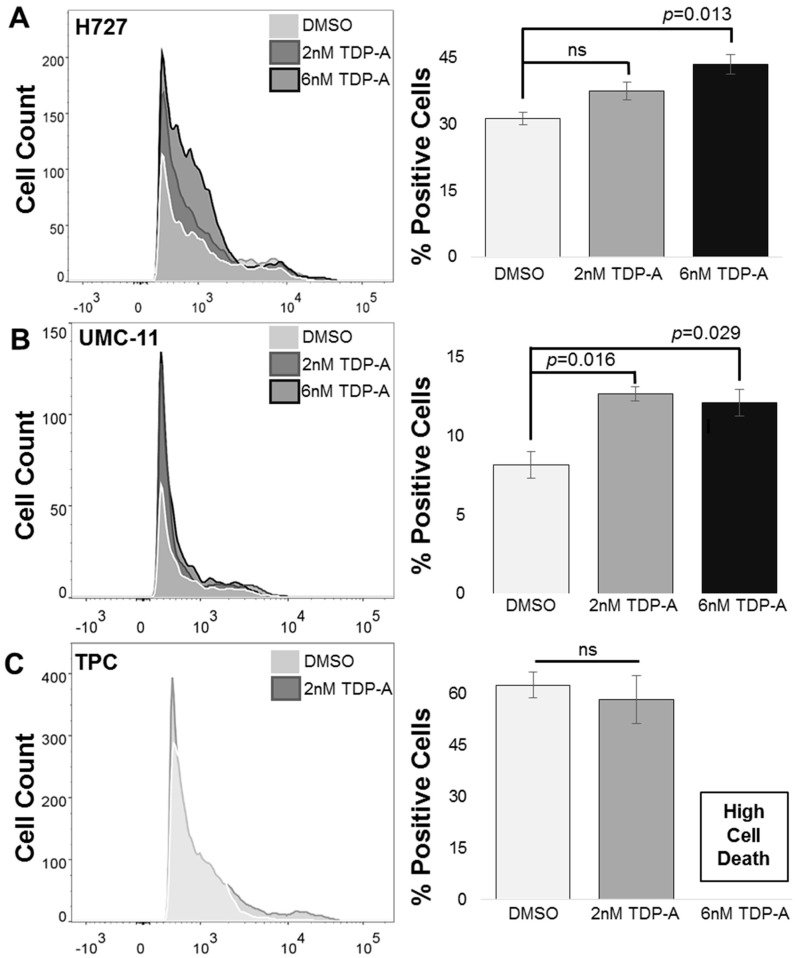
Increase in surface SSTR2 incidence. (**A**) The pulmonary carcinoid cell line H727 showed a significant increase in the percentage of cells expressing SSTR2 after being administered 6 nM of TDP-A (**B**) UMC-11, another pulmonary carcinoid cell line, showed significant increases in the percentage of SSTR2-positive cells after receiving either 2 nM or 6 nM of TDP-A (**C**) A papillary thyroid carcinoma cell line, TPC, did not show any change in SSTR2 expression after TDP-A administration. ns: not significant.

Fluorescence-activated cell sorting (FACS) of the pulmonary carcinoid cell line H727 in comparison to the papillary thyroid cancer cell line (TPC) confirmed the functional overexpression of SSTR2 expression after HDAC inhibitor treatment (Figure 4). There was a statistically significant difference between the H727 cells treated with DMSO as a control and a 6 nM dose of TDP-A as determined by one-way ANOVA F(2,6) = 9.959, *p* =0.012. A Bonferroni post hoc test revealed that the H727 cells treated with 6 nM TDP-A (mean ± SEM, 43.53 ± 3.96, *p* = 0.013) resulted in a significantly higher percentage of cells expressing SSTR2 on the cell surface compared to the control (DMSO) treatment (mean ± SEM, 31.27 ± 2.51). There was no significant difference between the control (DMSO) treatment and the 2 nM TDP-A treatment (mean ± SEM, 37.6 ± 3.47). Interestingly, the UMC-11 cell line showed lower overall expression of cell surface SSTR2 compared to H727, but still had a significant increase in the percentage of cells with detectable SSTR2 expression F(2) =10.904, *p* = 0.010. Specifically, UMC-11 cells treated with either 2 nM TDP-A (mean ± SEM, 8.16 ± 0.86) or 6 nM TDP-A (mean ± SEM, 12.1 ± 0.85) both had a significantly greater amount of cells expressing SSTR2 compared to cells treated with DMSO as a control. In the non-neuroendocrine cancer cell line (TPC), there was not a significant difference in cells treated with either control (DMSO) or 2 nM TDP-A, *t*(4) = 0.546, *p* = 0.614 as determined by a one-way ANOVA. The 6 nM dose of TDP-A was highly cytotoxic to TPC cells and therefore no viable cells were available for analysis.

### 2.3. Cell Uptake of [^68^Ga]DOTATATE PET/CT In Vitro

**Figure 5:**
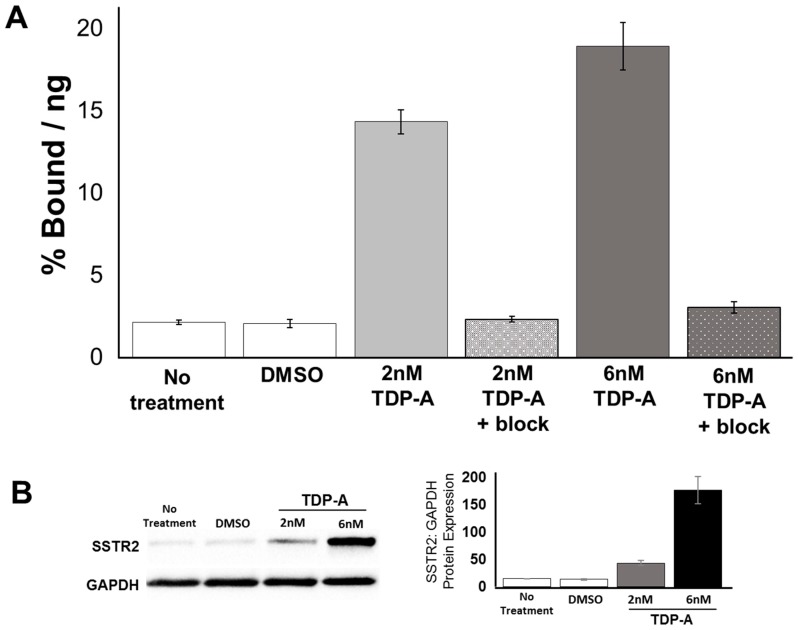
[^68^Ga]DOTATATE uptake in H727 cells treated with the HDAC inhibitor TDP-A. (**A**) The in vitro uptake of [^68^Ga]DOTATATE was significantly greater in H727 cells treated with either 2 nM or 6 nM of TDP-A for 48 h in comparison to non-treated cells and cells treated with DMSO as a control. Additionally, an unlabeled octreotide peptide was added to block the SSTR2 and showed no non-specific binding. (**B**) Western blot results with protein quantification data verifying an increase in SSTR2 expression after 24 h of treatment with both 2 nM and 6 nM TDP-A (Supplementary Figure S5).

A 48 h treatment with TDP-A had a significant effect on the uptake of [^68^Ga]DOTATATE in H727 cells, as determined by a one-way ANOVA, F(5) = 116.267, *p* < 0.001 (Figure 5A). H727 cells treated with 2 nM TDP-A (mean ± SEM, 14.33 ± 0.74) had a significantly higher percentage of [^68^Ga]DOTATATE uptake than cells left untreated (mean ± SEM, 2.13 ± 0.14, *p* < 0.001) or treated with DMSO as a control (mean ± SEM, 2.04 ± 0.24, *p* < 0.001). Likewise, H727 cells treated with 6 nM TDP-A (mean ± SEM, 18.91 ± 1.43, *p* < 0.001) had a significantly higher percentage of [^68^Ga]DOTATATE uptake than cells left untreated (mean ± SEM, 2.13 ± 0.14, *p* < 0.001) or treated with DMSO as a control (mean ± SEM, 2.04 ± 0.24, *p* < 0.001). Furthermore, the uptake was significantly higher in cells treated with 6 nM TDP-A than 2 nM TDP-A. Moreover, the percentage of [^68^Ga]DOTATATE uptake was measured when unlabeled octreotide peptide was added to block SSTR2 to test for non-specific binding in the population of cells that received either 2 nM or 6 nM TDP-A. In this experiment, the percentage of [^68^Ga]DOTATATE uptake was significantly reduced in cells treated with 2 nM TDP-A + block (mean ± SEM, 2.31 ± 0.17, *p* < 0.001) and cells treated with 6 nM TDP-A + block (mean ± SEM, 3.03 ± 0.35, *p* < 0.001) in comparison to the cells treated with the corresponding dose of TDP-A that not did receive the unlabeled octreotide peptide. The increase in the uptake of [^68^Ga]DOTATATE in cells treated with TDP-A was completely reduced when the unlabeled octreotide peptide was present. Therefore, this result indicates that the detected increase in [^68^Ga]DOTATATE uptake likely resulted from a higher density of SSTR2 on the cell surfaces. Additionally, a western blot was performed to verify that the expression of SSTR2 can be increased in H727 cells after a 24 h treatment with TDP-A (Figure 5B, Figure S5). The results show that after 24 h, SSTR2 expression increased nearly 3-fold after treatment with 2 nM TDP-A and approximately 12.5-fold after treatment with 6 nM TDP-A. This data supports the conclusion that the observed increase in the uptake of [^68^Ga]DOTATATE in H727 cells results from a greater incidence of SSTR2.

### 2.4. [^68^Ga]DOTATATE PET/CT Small Animal Imaging

**Figure 6:**
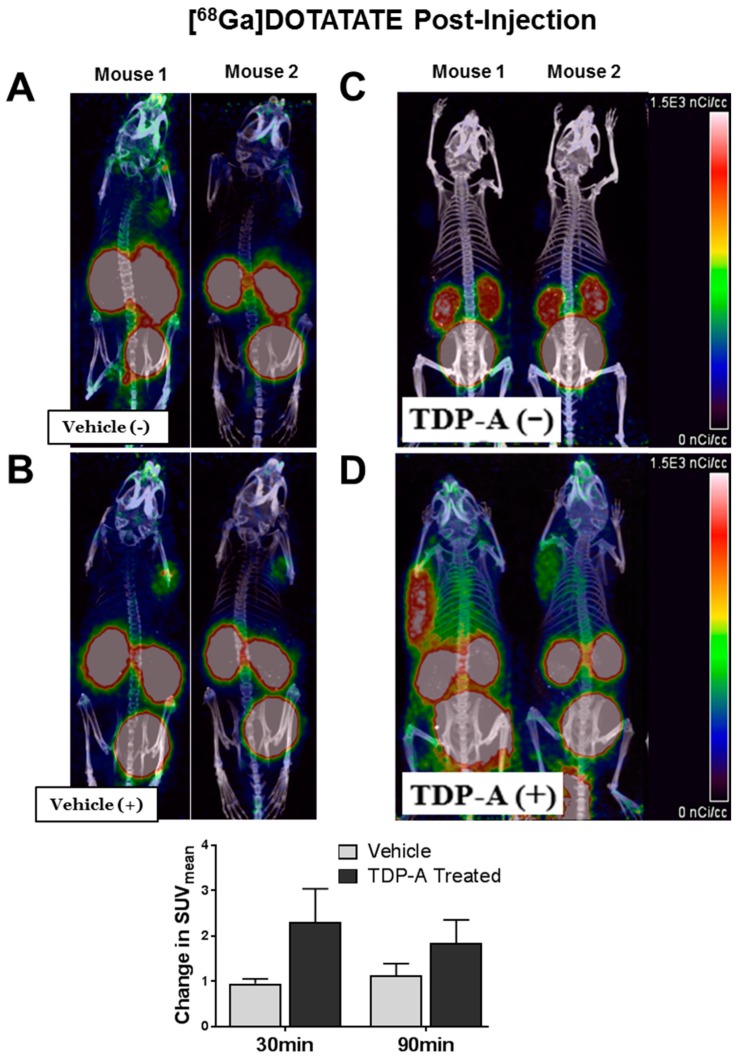
Mice bearing pulmonary NET xenografts and pretreated with TDP-A (+) demonstrated improved [^68^Ga]DOTATATE binding. (**A**) Two mice were imaged 30 min after [^68^Ga]DOTATATE administration before control treatment (Vehicle (−)) and then imaged again (**B**) 21.5 h after control treatment (Vehicle (+)). (**C**) Four mice were imaged 30 min after [^68^Ga]DOTATATE administration before TDP-A(−) treatment and then imaged again (**D**) 21.5 h after TDP-A(+) treatment.

Mice bearing pulmonary carcinoid xenografts (H727) demonstrated improved [^68^Ga]DOTATATE binding after HDAC inhibitor administration when imaged using PET/CT (Figure 6). Mice given the vehicle control treatment showed a minimal increase in [^68^Ga]DOTATATE binding (Figure 6A,B). However, mice treated with TDP-A displayed an evident increase in [^68^Ga]DOTATATE binding (Figure 6C,D). The mice that received HDAC inhibitor administration had an average increase in the standard uptake value (SUV) of 2.31 ± 0.73 (mean ± SEM) at 30 min post-[^68^Ga]DOTATATE injection and 1.84 ± 0.52 (mean ± SEM) at 90 min post-[^68^Ga]DOTATATE injection. On the other hand, the vehicle-treated group had average SUV change of only 0.96 ± 0.10 (mean ± SEM) at 30 min post-[^68^Ga]DOTATATE injection and 1.14 ± 0.25 (mean ± SEM) at 90 min post-[^68^Ga]DOTATATE injection, indicating improved detection of pulmonary carcinoid subcutaneous xenografts. There was no statistical difference between the absolute uptake values likely due to the disparity in the individual mouse tumor uptake and tumor sizes.

## 3. Discussion

In this study, we have illustrated that treating pulmonary carcinoids with five different HDAC inhibitors can result in the overexpression of the targetable surface receptor SSTR2. Pulmonary carcinoids reportedly have variable SSTR2 expression, with a SSTR2A subtype frequency of 72% [13,23], making these tumors particularly difficult to image and treat using common NET-specific techniques such as PET/CT with radiolabeled somatostatin analogs that specifically bind to SSTR2. Consequently, these patients would greatly benefit from a technique that could help identify and track previously undetectable primary and metastatic pulmonary carcinoids. In turn, such advancement would not only create a more comprehensive picture of the disease in a patient, but also establish a protocol for more precise imaging and targeted therapies [24].

Our approach to improve pulmonary carcinoid detection was centralized around the methodology that standard and widely accepted techniques for NET imaging and treatment could be improved to increase overall efficacy. In this study, we used HDAC inhibitors to drive an increase in SSTR2 incidence and density. Traditionally, the HDAC protein family plays a key role in the regulation of the cell cycle, cell differentiation, apoptosis, migration and invasion, and angiogenesis by increasing histone acetylation; while HDAC inhibitors can nonselectively inhibit all or multiple members in the family to produce an anti-cancer effect in various cancers including NETs [18,19,20,21,22]. This study suggests that HDAC inhibitors may have an additional theranostic property in pulmonary carcinoids by increasing SSTR2 expression. As previously reported, there is evidence that the interaction of somatostatin and its’ analogs with somatostatin receptors has an antiproliferative action and can induce apoptosis, in addition to inhibiting tumorigenic hormone and growth factor secretion [8,9]. Furthermore, there is evidence that patients with low tumor SSTR expression have poor prognoses [15,25,26]. Therefore, one would expect that an increase in SSTR2 could improve patient outcome from a molecular level. However, one study challenges this idea through their finding that SSTR expression was not associated with overall or event-free survival in a study consisting of 102 patients with lung carcinoids [27].

Herein, we tested three FDA approved and two non-FDA approved HDAC inhibitory compounds. Our in vitro studies showed that TDP-A, a naturally derived but non-FDA approved compound was capable of increasing the expression of SSTR2 at the protein and mRNA level. Using small animal PET/CT imaging with [^68^Ga]DOTATATE, the administration of TDP-A increased tumor uptake and tumor-to-background ratio and thereby converted almost undetectable H727 xenografts into detectable tumors. To the author’s knowledge, there is no data available regarding the clinical dosing of TDP-A as it not FDA approved. For the in vivo study, a single dose of TDP-A was administered at the maximum tolerated dose (MTD) of 12.5 mg/kg, as previously tested in mice. The same study shows a therapeutic effect of TDP-A when mice received five intravenous injections every 4 days of the MTD [28]. However, the study described herein does not aim for a therapeutic effect, but instead a non-toxic dose to increase SSTR2 expression.

Moreover, a higher incidence of SSTR2 would localize radiolabeled analog binding while increasing uptake to ameliorate nuclear medicine approaches to NET imaging and therapy. This method is particularly advantageous to patients with pulmonary carcinoids because these tumors often have variable SSTR2 expression [23,29], in which we observed both in vitro and in vivo.

These results could be translatable to a clinical setting because four HDAC inhibitors are currently FDA approved [30] and radiolabeled somatostatin analog-based NET imaging is standardized and FDA-approved for pulmonary carcinoids. The administration of a HDAC inhibiting drug could increase SSTR2 incidence to allow for the detection of hidden primary and metastatic nodules with minimal SSTR2 expression, benefiting patients who have failed or are ineligible for current technologies.

Our approach could be improved as it was limited by the use of only two pulmonary carcinoid lines and five HDAC inhibitors. Universally, there is a limited amount of neuroendocrine cancer models, especially pulmonary carcinoids, available for study. It would be advantageous to screen more HDAC inhibitory compounds to expand the availability of drugs for patient administration.

Overall, the use of this methodology for the molecular upregulation of SSTR2 expression could improve target binding for precise imaging of pulmonary carcinoids, which previously have been difficult to image and treat. This technique could be translated to the clinic to benefit patients with marginal or diminished SSTR2 expression with other types of NETs.

## 4. Materials and Methods

All studies described were reviewed and approved by the Institutional Biosafety Committee at the University of Alabama at Birmingham under the project number 16-203 and the IACUC animal protocol approval code 20244.

### 4.1. Cell Culture

This study used human fibroblast cells (917 and WI38 cells), aggressive thyroid cancer cell lines (TPC, FTC236 and Hth7), and NET cell lines (medullary thyroid cancer: TT and MZ-CRC-1 cells, pancreatic NE cancer: BON-1, and pulmonary carcinoid: H727 and UMC-11). Cell lines were maintained as previously described (BON-1 [28], MZ-CRC-1 and TT [15], H727 [31], TPC, FTC236, and Hth7 [32]). The pulmonary carcinoid cell line UMC-11 were grown in RPMI 1640 containing glutamine (Invitrogen Life Technologies, Carlsbad, CA, USA) and supplemented with 10% fetal bovine serum, 100 IU/mL penicillin, and 100 μg/mL streptomycin. The lung fibroblast cell line WI-38 was grown in MEM medium (Invitrogen Life Technologies, Carlsbad, CA, USA) containing Earl salts, glutamine, and phenol red, in addition to 10% fetal bovine serum, 1% non-essential amino acids, and 1% sodium pyruvate. 917, a foreskin fibroblast cell line, was grown in MEM medium (Invitrogen Life Technologies, Carlsbad, CA, USA) with 10% fetal bovine serum, 1% non-essential amino acids, sodium bicarbonate, 0.05 M tricine, and 100 IU/mL penicillin, and 100 μg/mL streptomycin. All cells were grown at 37 °C in a humidified atmosphere containing 5% CO2. Cells were treated for either 24 or 48 h with either DMSO as a control or the following concentrations of HDAC inhibitors: 2 nM or 6 nM TDP-A, 2 nM or 6 nM FK228, 1 µM or 3 µM AB3, 1 µM or 3 µM SAHA and 1mM or 4 mM VPA.

### 4.2. Real Time Quantitative PCR

Total RNA was isolated from the treated cells 24 h after treatment with the HDAC inhibitor or DMSO using a RNeasy Plus Mini kit (Qiagen, Hilden, Germany). RNA concentrations were determined using the NanoDrop 1000 spectrophotometer (Thermo Scientific, Waltham, MA, USA). Complementary DNA was synthesized from 2μg of total RNA using iScript cDNA Synthesis Kit (Bio-Rad, Hercules, CA, USA). The real-time quantitative PCR was performed in triplicate on CFX Connect Real-Time PCR Detection System (Bio-Rad). The PCR primer sequences for: SSTR forward primer: 5′GAG AAG AAG GTC ACC CGA ATG G 3′, SSTR reverse primer: 5′ TTG TCC TGC TTA CTG TCA CTC CGC 3′, GAPDH forward primer: 5′ ACC TGC CAA ATA TGA TGA C 3′, GAPDH reverse primer: 5′ ACC TGG TGC TCA GTG TAG 3′. Target gene expression was normalized to GAPDH and the ΔΔCt method was used to calculate relative gene expression. Error bars show the Standard Error of the Mean (SEM).

### 4.3. Western Blot Analysis

Basal SSTR2 expression was determined in the cell lines: 917, WI38, TPC, FTC236, Hth7, TT, MZ-CRC-1, BON-1, H727 and UMC-11. Cells were treated with DMSO as a control or with different concentrations of the HDAC inhibitory drugs: 2 nM or 6 nM TDP-a, 2 nM or 6 nM FK228, 1 µM or 3 µM AB3, 1 µM or 3 µM SAHA and 1 mM or 4 mM VPA. After 48 h, cell lysates and cell surface proteins were prepared. The cell surface proteins were isolated using the Pierce Cell Surface Protein Isolation Kit (Thermo Scientific). Lysate protein concentrations were quantified by BCA Protein Assay Kit (Thermo Scientific) and cell surface proteins were quantified by Pierce 660 nm Protein Assay (Thermo Scientific). The protein samples were denatured and then resolved by a 4–15% Criterion TGX gradient gel (Bio-Rad) electrophoresis, transferred onto nitrocellulose membranes (Bio-Rad), blocked in milk (1× PBS, 5% dry skim milk and 0.05% Tween-20) for 1 h at 4 °C, and incubated with anti-SSTR2 primary antibody (SSTR2 antibody (A-8): sc-365502, Santa Cruz, Dallas, TX, USA) with 1:500 dilution overnight at 4 °C. The membrane was then incubated with the secondary antibody (anti-mouse HRP linked antibody 1:1000, Cell Signaling Danvers, MA, USA) for 2 h at room temperature. The protein bands were detected by Luminata Forte Western HRP Substrate (Millipore, Burlington, MA, USA). Expression levels of GAPDH was used as a loading control for lysates and the expression levels of the calcium pump pan-PMCA ATPase (ab2825, Abcam, Cambridge, UK) was used as a loading control for membrane fractions. Protein expression relative to GAPDH as a loading control was quantified using ImageJ software (NIH, Bethesda, MD, USA).

### 4.4. Flow Cytometry

H727, UMC-11 and TPC cells were treated with either DMSO, 2 nM TDP-A, or 6 nM concentration of TDP-A for 48 h. After treatment, cells were stained for 1 h at 37 °C with an anti-SSTR2 antibody (Cat#MAB4224, Novus Biologicals, Centennial, CO, USA) labeled with Cy5.5 fluorophore (Lumiprobe, Hunt Valley, MD, USA) at a concentration of 1 µg. After staining, cells were resuspended in flow buffer (0.5% BSA in sterile PBS) then run on a flow cytometer (LSRII, BD Biosciences, San Jose, CA, USA) to detect the presence of Cy5.5 signal via Alexa Fluor 700 laser. Each experiment was done in triplicate. Data were analyzed using FlowJo V5.0 (TreeStar, Inc., Ashland, OR, USA).

### 4.5. In Vitro [^68^Ga]DOTATATE PET/CT Uptake

An in vitro study was performed to measure changes in [^68^Ga]DOTATATE uptake between H727 cells left untreated, treated with DMSO as a control, or treated with either 2 nM or 6 nM of the HDAC inhibitor TDP-A. This experiment was performed in two sets of 12-well plates for each condition. Cells were incubated with either DMSO, 2 nM TDP-A, 6 nM TDP-A, or untreated media for 48 h. For measuring radiopeptide uptake, [^68^Ga]DOTATATE labeled at specific activity of 215 µCi/µg, with a final concentration of 8 µg/mL (5.3 µM, MW = 1502.3 g/mol) was used. The binding study was done in six replicates at 37 °C for 2 h under 5% CO_2_ using a peptide chelate concentration of 10 nM. In another set of wells, 40 µM of unlabeled octreotide peptide was added to block SSTR2 to check for non-specific binding. After incubation, the cells were washed three times with ice-cold PBS and lysed with 0.4 M NaOH. The activity associated with the cells was measured using a gamma counter and protein content evaluated using the BCA assay kit. The data is presented as % Bound per ng of protein = activity associated with cells per total activity added per amount of protein in the lysed cells.

### 4.6. Small Animal [^68^Ga]-DOTATATE PET/CT Imaging

Immunocompromised male Nu/Nu mice (Jackson Laboratories, Bar Harbor, ME, USA) were subcutaneously injected with H727 cells and xenografts developed to a palpable size in three weeks. Two groups of mice were imaged before (basal images) and after injection of vehicle or TDP-A. Small animal PET images were acquired on a Sophie GNEXT scanner following tail vein injection of 4.3–5.2 MBq (115–140 µCi) of [^68^Ga]DOTATATE. Static scans were collected at 30 min and 90 min post-injection. A dose of 12.5 mg/kg TDP-A was administered to mice receiving TDP-A treatment while non-treated mice received equal volume of TDP-A dissolution vehicle (10% ethanol, 60% PEG, 30% PBS) after basal small animal PET images were acquired. Then, mice were imaged again 24 h after vehicle or TDP-A injection. PET and CT images were acquired on a Sofie GNEXT PET/CT scanner. The CT images were reconstructed using a Modified Feldkamp Algorithm. The PET images were reconstructed using a 3D-OSEM (Ordered Subset Expectation Maximization) algorithm (24 subsets and 3 iterations), with random, attenuation and decay correction. Regions of interest were drawn and the mean and maximum standard uptake values (SUVs) for tumors were determined using the formula: SUV = [(MBq/mL) × (animal wt. (g))/injected dose (MBq)].

### 4.7. Statistical Analysis

All statistical analyses were performed using SPSS (IBM Corp. Released 2017. IBM SPSS Statistics for Windows, Version 25.0. Armonk, NY, USA: IBM Corp.). Significance was determined using a one-way ANOVA followed by a Tukey *post hoc* test, unless stated otherwise. Data were normally distributed. All data is expressed as mean ± Standard Error Mean (SEM) unless stated otherwise. Quantitative real-time PCR and flow cytometry experiments were done triplicate. *p* values < 0.05 were statistically significant.

## 5. Conclusions

This study describes a potential method of increasing the membranous density of SSTR2 in pulmonary neuroendocrine tumors through the use of HDAC inhibitory compounds. A higher incidence of SSTR2 on the cell membrane could enhance the efficacy of imaging techniques and therapeutics that target SSTR2.

## Supplementary Materials

The following are available online at https://www.mdpi.com/2072-6694/11/6/767/s1, Figure S1: Complete western blot image corresponding to Figure 1A showing all bands and molecular weight markers for (A) SSTR2 and (B) GAPDH, which served as a loading control, Figure S2: Complete western blot image corresponding to Figure 1B showing all bands and molecular weight markers for (A) SSTR2 and (B) GAPDH, which served as a loading control, Figure S3: Complete western blot image corresponding to Figure 3A showing all bands and molecular weight markers for SSTR2 (top image) and GAPDH, which served as a loading control (bottom image) for the cell lines: (A) H727 (B) UMC-11 (C) TT (D) MZ, Figure S4: Complete western blot image corresponding to Figure 3C showing all bands and molecular weight markers for (A) SSTR2 and after the membrane was stripped and probed for (B) the calcium ion pump, which served as a loading control, Figure S5: Complete western blot image corresponding to Figure 5B showing all bands and molecular weight markers for (A) SSTR2 and (B) GAPDH, which served as a loading control.
