# Supplementary material for: Pulmonary Carcinoid Surface Receptor Modulation Using Histone Deacetylase Inhibitors
Source: Cancers (Basel). 2019 Jun 3;11(6):767. doi: 10.3390/cancers11060767 (PMC6627607; doi:10.3390/cancers11060767)
Supplement: Supplementary file 1 [file cancers-11-00767-s001.pdf]

cancers

Supplementary Materials: Pulmonary Carcinoid Surface Receptor Modulation Using Histone Deacetylase Inhibitors

Rachael E. Guenter, Tolulope Aweda, Danilea M. Carmona Matos, Jason Whitt, Alexander W. Chang, Eric Y. Cheng, X. Margaret Liu, Herbert Chen, Suzanne E. Lapi and Renata Jaskula-Sztul

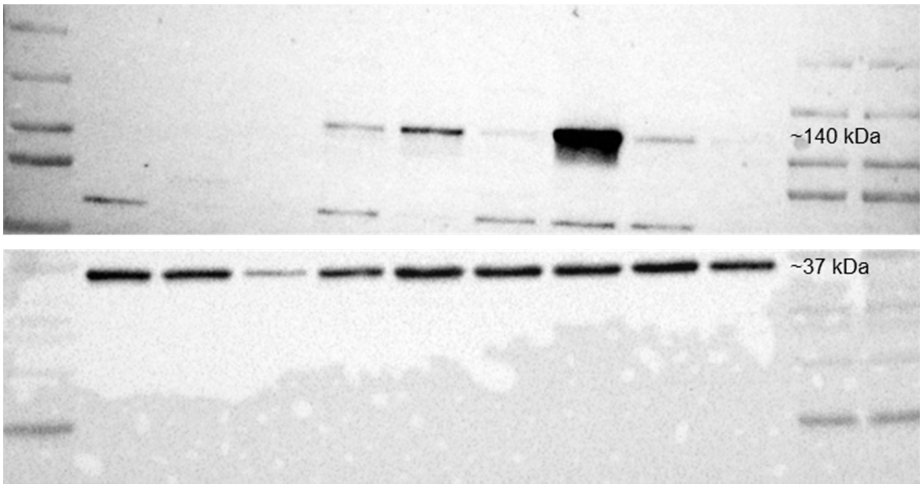

Figure S1. Complete western blot image corresponding to Figure 1A showing all bands and molecular weight markers for (**A**) SSTR2 and (**B**) GAPDH, which served as a loading control.

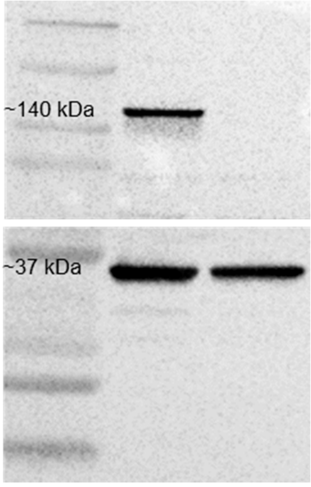

Figure S2. Complete western blot image corresponding to Figure 1B showing all bands and molecular weight markers for (**A**) SSTR2 and (**B**) GAPDH, which served as a loading control.

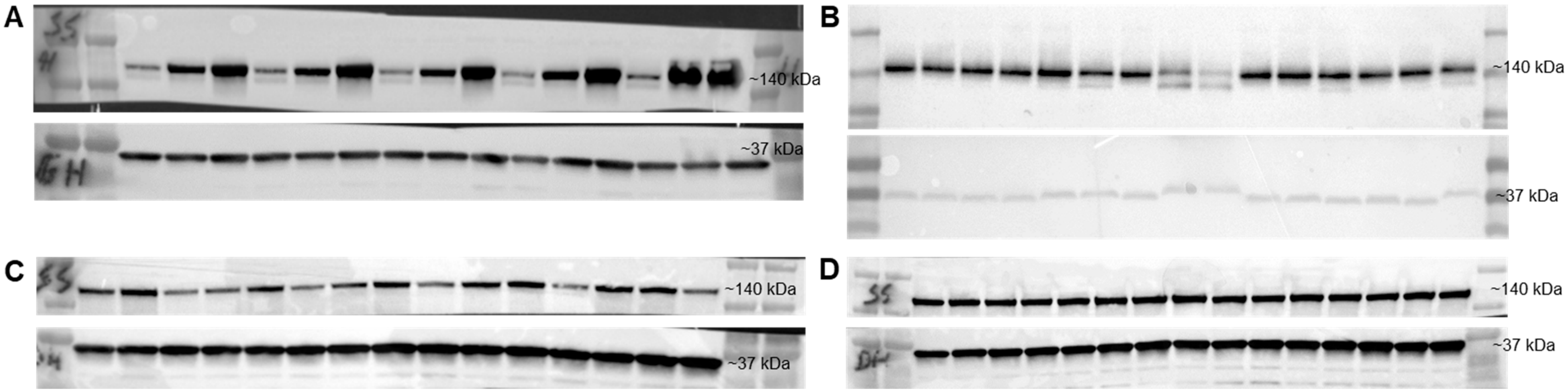

Figure S3. Complete western blot image corresponding to Figure 3A showing all bands and molecular weight markers for SSTR2 (top image) and GAPDH, which served as a loading control (bottom image) for the cell lines: (**A**) H727 (**B**) UMC-11 (**C**) TT (**D**) MZ.

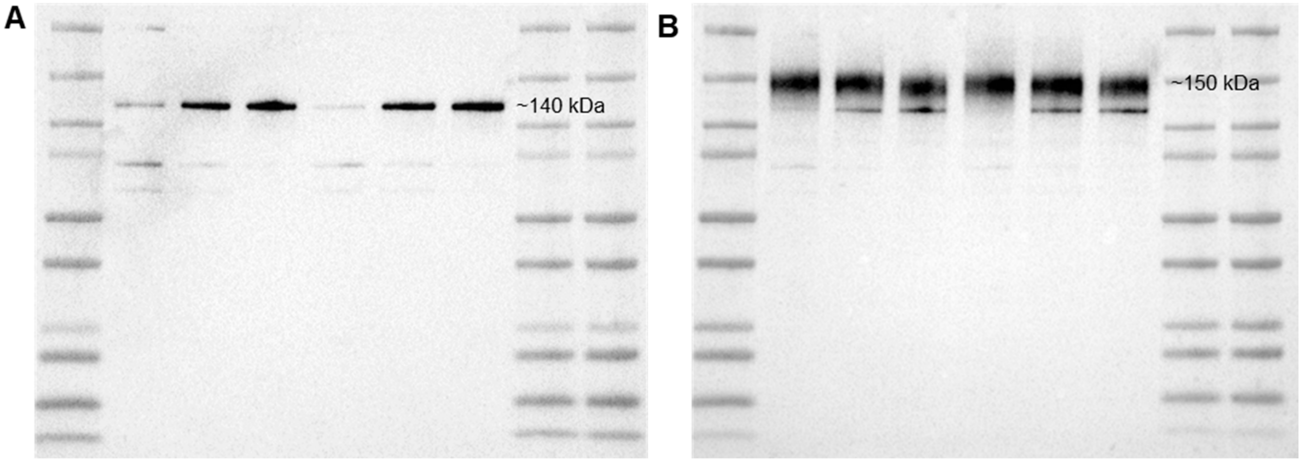

Figure S4. Complete western blot image corresponding to Figure 3C showing all bands and molecular weight markers for (**A**) SSTR2 and after the membrane was stripped and probed for (**B**) the calcium ion pump, which served as a loading control.

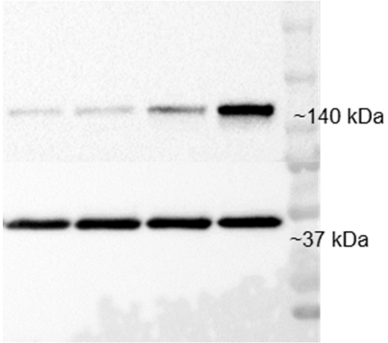

Figure S5. Complete western blot image corresponding to Figure 5B showing all bands and molecular weight markers for (**A**) SSTR2 and (**B**) GAPDH, which served as a loading control.

© 2019 by the authors. Licensee MDPI, Basel, Switzerland. This article is an open access article distributed under the terms and conditions of the Creative Commons Attribution (CC BY) license (http://creativecommons.org/licenses/by/4.0/).
